# Toward an Objective Diagnostic Test for Bacterial Cellulitis

**DOI:** 10.1371/journal.pone.0162947

**Published:** 2016-09-22

**Authors:** Daniel J. Pallin, Lynn Bry, Richard C. Dwyer, Adam D. Lipworth, Donald Y. Leung, Carlos A. Camargo, Thomas S. Kupper, Michael R. Filbin, George F. Murphy

**Affiliations:** 1 Department of Emergency Medicine, Brigham and Women’s Hospital, Boston, MA, United States of America; 2 Department of Pathology, Brigham and Women’s Hospital, Boston, MA, United States of America; 3 Department of Dermatology, Brigham and Women’s Hospital, Boston, MA, United States of America; 4 Department of Pediatrics, National Jewish Medical Health, Denver, CO, United States of America; 5 Department of Emergency Medicine, Massachusetts General Hospital, Boston, MA, United States of America; University of Ulster, UNITED KINGDOM

## Abstract

**Background:**

Prior studies repeatedly showed that cultures of skin lesions diagnosed as "cellulitis" are usually negative. However, lack of a gold standard for diagnosis (against which culture might be judged) and failure to assess the human immune response are important limitations of prior work. In this pilot study, we aimed to develop a criterion standard for research on bacterial cellulitis, to evaluate the sensitivity of procalcitonin for bacterial cellulitis, and to use gene expression analysis to find other candidate diagnostic markers.

**Methods:**

We classified lesions via biopsies, 16s rRNA gene detection, culture, and histopathology. We quantified procalcitonin expression in blood. We also used Nanostring technology to quantify transcription of immunomodulators that may distinguish cases from inflamed controls.

**Results:**

Of 28 participants, 15 had a clinical diagnosis of cellulitis, six had a diagnosis of non-infectious dermatitis, and seven were normal volunteers. Of the “cellulitis” patients, three (20%) had pathogens isolated, and were designated confirmed cases. Procalcitonin was undetectable in all three. HLA-DQA1 was expressed 34-fold more in confirmed cases vs. controls (fold change of geometric mean). Heat maps depicting multiplex gene expression analysis revealed a distinct profile of gene expression in confirmed cases relative to comparators.

**Conclusions:**

Most “cellulitis” patients had microbiologically-negative biopsies. Procalcitonin was undetectable, and HLA-DQA1 elevated, in confirmed bacterial cases. Multivariable transcriptomic profiling results supported our algorithm’s ability to identify patients with true bacterial cellulitis. A larger sample may allow discovery of an immunological signature capable of distinguishing bacterial cellulitis from its mimics in clinical practice.

## Introduction

An editorial in *Clinical Infectious Diseases* compared cellulitis to pornography,[1] because both are recognized subjectively, based on the standard enunciated by U.S. Supreme Court Justice Potter Stewart: "I know it when I see it." The subjectivity of the diagnosis of cellulitis has been the topic of several investigations, but these studies relied on expert opinion, which is also subjective, as the reference standard. These studies found that specialists deem generalists' diagnosis of cellulitis to be wrong in up to 74% of cases.[2–4] Prior studies have also been limited by the lack of a control group, and lack of assessment of the human immune response.

The diagnosis of “cellulitis” is very common, and it is a frequent reason for antibiotic utilization and hospitalization. Skin infection diagnoses account for 6.3 million office visits and 3.8 million emergency department visits per year in the US alone.[5–7] Roughly four out of five acute care visits with diagnosis of bacterial skin infection are for cellulitis, with the minority, one in five, being for abscess.[8] There are approximately 900,000 U.S. hospitalizations each year for skin infections.[9] Healthcare expenditures for skin infections total >$10 billion per year.[5,8,10] If many or most of these cases are wrongly diagnosed, then we are prescribing antibiotics and hospitalizing patients unnecessarily quite frequently.

The logical objective test for bacterial cellulitis would be culture for pathogens, but blood cultures are positive in only 4%, and, even with culture of biopsied tissue, an organism can be identified in <30%.[11–13] A review of 16 studies from 1966–2007 using needle aspiration or biopsy to identify pathogens in cellulitis found that only 16% of cellulitis cases had positive cultures.[13] The inability of cultures to identify pathogens suggests that either pathogens are not present or that culture is the wrong test. Culture-independent biomarkers are needed, and in order to discover such biomarkers, we need an objective criterion standard that can classify research participants as having bacterial cellulitis vs. a non-bacterial inflammatory skin condition (i.e. a cellulitis mimic).

We have developed an algorithm that might serve as a gold standard, objectively separating lesions into bacterial vs. nonbacterial skin inflammation. We apply this algorithm to a pilot sample of 28 subjects with diverse clinical presentations. Against this gold standard, we test the sensitivity of elevated procalcitonin, which has been reported to be specific for bacterial infection.[14,15] We present methods for the use of transcriptomics to search for a host immunological signature that might assist future researchers (and clinicians) to distinguish cases from controls.

## Methods

**Fig 1:**
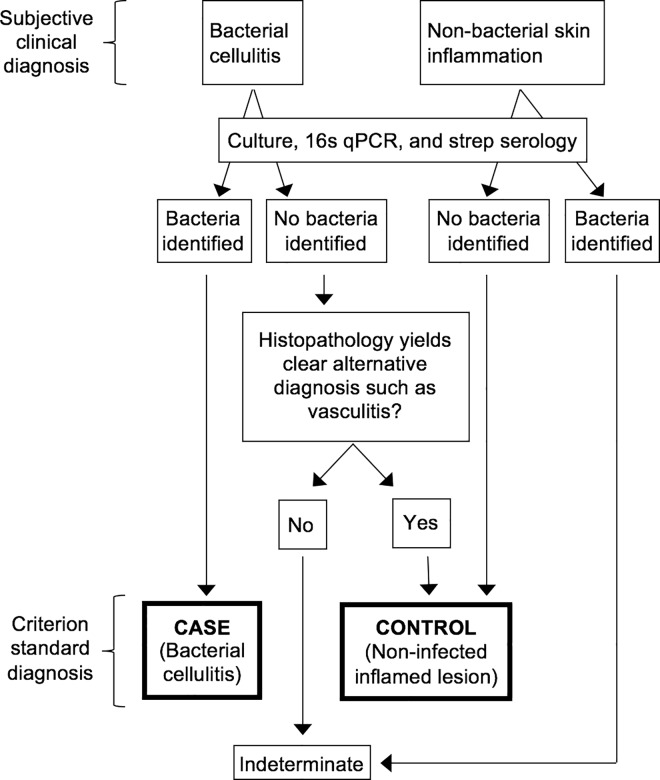
Algorithm for Identification of Confirmed Cellulitis Cases and Controls. Please see the text for an explanation of the application of this algorithm.

We propose the algorithm shown in Fig 1 as a gold standard for the diagnosis of cellulitis in research studies. Its novelty is that it uses only verified bacterial cellulitis and verified sterile inflamed controls for biomarker identification, excluding indeterminate presentations from the discovery effort. Whereas prior cellulitis investigations have pooled the culture-positive and culture-negative participants under the subjective umbrella diagnosis of “cellulitis,” our algorithm separates them, and divides participants into those that are classifiable by microbiology and histopathology vs. those that are not classifiable. Our approach is also novel in that other studies of cellulitis have been uncontrolled case series, while we compare cases to controls.

Only those who are classifiable are considered cases or controls. The *cases* are those who have a clinical diagnosis of cellulitis and microbiologically-proven infection. The *controls* are (a) participants with a clinical diagnosis of non-infectious dermatitis and no microbiological evidence of bacteria; or (b) participants originally thought to have bacterial cellulitis but found to have sterile lesions and to have histopathology diagnostic of another entity such as vasculitis or hypersensitivity reaction (Fig 1).

Those that are not classifiable (*indeterminate* patients) are (a) participants with a clinical diagnosis of cellulitis but sterile lesions and non-diagnostic pathology; or (b) those with a clinical diagnosis of non-infectious dermatitis whose lesions are found to harbor bacteria. The utility of this approach is that the "classifiable" participants can be used as cases and controls in a search for biomarkers of infection. Identified biomarkers can then be used to classify indeterminate patients, and eventually developed into clinical diagnostic tests.

We applied the algorithm to a pilot sample of participants from three clinical diagnostic groups, all recruited from a Boston emergency department, with clinical diagnoses made by emergency physicians in routine practice. Diagnosis was made by practicing emergency physicians relying upon clinical judgment, without any structured diagnostic criteria; robustness of classification was insured by application of the algorithm shown in Fig 1, which only allows lesions whose clinical diagnosis and laboratory diagnosis meet the definition of case or control to be classified as such. There were three clinical diagnostic categories: (1) diagnosed with cellulitis and prescribed antibiotics, (2) diagnosed with a non-infectious dermatitis and not prescribed antibiotics, and (3) normal volunteers. The only inclusion criterion was fitting into one of the above categories. Exclusion criteria were having the skin lesion above the clavicle or on the hand, foot, or genitals (given the need for a research skin biopsy); concern that the biopsy might not heal well due to location or peripheral vascular disease; and any other known bacterial infection. Every participant underwent skin biopsies and blood sampling. The above three clinical diagnostic groups were analyzed according to the algorithm in Fig 1, and then categorized into one of four groups: (1) confirmed infected cases, (2) confirmed sterile inflamed controls, (3) indeterminate lesions, or (4) normal volunteers. In our transcriptomic investigation, we sought biomarkers capable of distinguishing cases from controls (mimics) using groups 1 and 2. We then compared the cases in group 1 to each of the other three groups.

All blood and biopsy samples for procalcitonin testing, transcriptomic analysis, histopathology, and microbiology, were obtained during the emergency department visit at which enrollment occurred.

Biopsies were taken from skin that was cleaned with 3 alcohol wipes, without betadine or chlorhexidine. The participants with lesions had a 3 mm punch biopsy of the lesion for histopathology and a 2 mm punch biopsy of the lesion for microbiology. They also had a 3 mm punch biopsy of contralateral normal skin for histopathology and a 2 mm punch biopsy of contralateral normal skin for microbiology. Normal volunteers had a 3 mm punch biopsy for histopathology and a 2 mm punch biopsy for microbiology.

The 3 mm biopsies were fixed in formalin and embedded in paraffin. A senior histopathologist (GFM) blinded to clinical diagnosis studied sections prepared with Gram's and hematoxylin and eosin stains. Also, RNA was isolated from the paraffin section and subjected to Nanostring analysis. Nanostring technology uses bar-coded probes to quantify messenger RNA in a reliable multiplex fashion[16] and is a reference standard used to validate other assays.[17,18] Although Nanostring technology is in wide use in cancer biology, there is little precedent for its use in infectious disease biology, and no precedent for its use in skin infections. We used the GX Immunology V2 codeset, which quantifies mRNA from 594 immunomodulator genes.[19] We also included a custom probe for CALCA, the gene encoding procalcitonin. Analysis involved isolation of 100 ng RNA and quantification of the number of mRNA molecules present in this sample.

**Fig 2:**
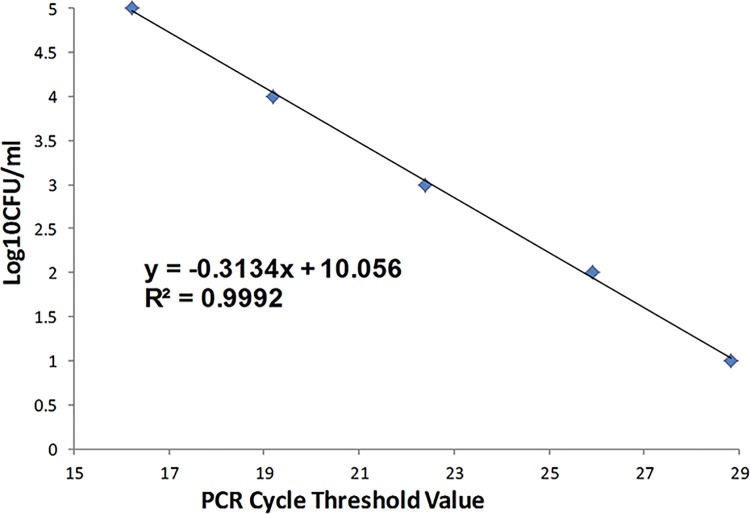
Assessment of qPCR Sensitivity for Bacteria Using *S*. *epidermdis*. We assessed the sensitivity of quantitative PCR for detection of bacteria via amplification of the universal bacterial 16s ribosomal RNA gene, using DNA purified from germ free mice skin tissue spiked with serial 10-fold dilutions of quantified *Staphylococcus epidermidis*. The assay's sensitivity was at or better than 10 colony-forming units per mL, with no evidence of decreasing sensitivity at lower levels.

The 2 mm biopsies were frozen within 1 hour. Later, they were homogenized, and from the homogenate, a standard culture was performed for bacteria and fungi, and quantitative PCR was performed with probes targeting the universal bacterial 16s ribosomal RNA gene. We assessed the sensitivity of this method for detection of bacteria using DNA purified from germ free mice skin tissue spiked with serial 10-fold dilutions of quantified *Staphylococcus epidermidis*. The assay's sensitivity was at least 10 colony-forming units per mL (Fig 2). Human GAPDH was amplified as an internal control (Forward: TTG CCA TCA ATG ACC CCT TCA; Reverse: CGC CCC ACT TGA TTT TGG A).

Serum was collected and procalcitonin quantified using the VIDAS B.R.A.H.M.S Procalcitonin^®^ assay (Biomerieux, Inc., Durham, NC).[20] For Nanostring analysis of blood, we used a PAXGene RNA isolation tube to collect blood and then isolated 100 ng RNA for testing with the GX Immunology V2 codeset, which quantifies mRNA from 594 immunomodulator genes.[19] We also included a custom probe for CALCA, the gene encoding procalcitonin. To identify any promising *individual* candidate markers to distinguish between cases and controls, we sorted all of the markers according to the expression fold-change (geometric mean) in cases vs. controls. Below we report the marker with the highest fold-change in geometric mean expression in cases and controls, and report the p-value calculated from a t-test of the mean of the log2-transformed count of mRNA molecules by group.

**Fig 3:**
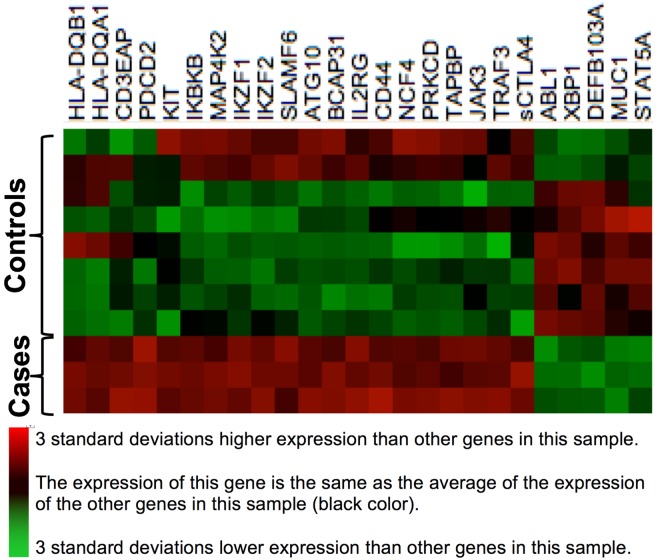
Heat Map of a Derivation Set, Comprised of Verified Cases and Inflamed Controls. This heat map, built on agglomerative clustering with linkage by mean and a Euclidian distance metric, shows a comparison of log(2)-transformed normalized gene expression data from blood. Rows represent individual genes, the symbols of which are shown at right. Columns represent individual participants in our study. Colors represent the z-scores of the counts of mRNA molecules per 100 ng RNA in blood; *red* represents a high ratio of expression relative to other genes in that participant, and *green* represents a low ratio of expression relative to other genes in that participant. The left three columns are confirmed bacterial cellulitis cases, and have high expression of genes HLA-DQB1 through sCTLA4, and low expression of genes ABL1 through STAT5A. All of the other columns are inflamed controls, as defined in the text.

**Fig 4:**
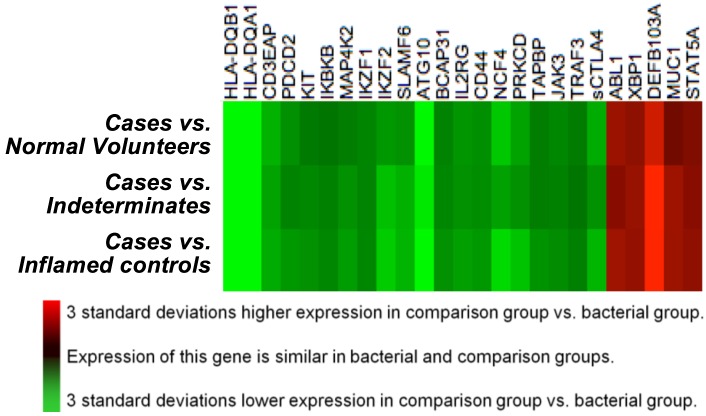
Comparison of Gene Expression Patterns in Verified Bacterial Cases versus Three Comparison Groups. This heat map, built on agglomerative clustering with linkage by mean and a Euclidian distance metric, shows a comparison of log(2)-transformed normalized gene expression data from blood. Rows represent individual genes, the symbols of which are shown at right. Each column displays a comparison of gene expression in bacterial cases (B) vs. one of three comparator groups: sterile inflamed controls (ic), indeterminate participants (ind), and normal volunteers (v). Qualitatively, this heat map reveals that the cases differ in a similar way from all three comparison groups. This, in turn, suggests that the cases–which had confirmed bacterial cellulitis based on biopsy–could be detectable by transcription profiling.

To identify a *panel* of biomarkers that might be accurate for identification of bacterial cellulitis, we ranked immunomodulators according to their p-values, as calculated above. We then selected all markers with a p-value ≤0.01. We display the expression of the resulting 25 genes using heat maps. Our eventual plan is to use lasso logistic regression[21] to generate a *quantitative* panel of markers with high discriminatory value. However, in the present investigation, the sample size was too small for such analysis, given the large number of predictors, and therefore we restricted ourselves to a qualitative analysis via inspection of heat maps (explained further in Results). The heat maps were generated using agglomerative clustering of Z score transformed gene expression ratios of cases vs. inflamed controls, with linkage by mean, and the Euclidean distance metric. The first heat map was generated using the verified cases and inflamed controls (Fig 3), and the second (Fig 4) compares gene expression in cases to gene expression in three comparison groups: inflamed controls, indeterminates, and normal volunteers.

The Partners Healthcare Human Subjects Research Committee approved the protocol. All participants gave written informed consent. All analyses were conducted using nSolver software (Nanostring Technologies), which was also used to perform agglomerative clustering to generate heat maps.

## Results

We enrolled 28 eligible participants (Table 1). These comprised 15 patients diagnosed with cellulitis and prescribed antibiotics, six diagnosed with non-infectious dermatitis and not prescribed antibiotics, and seven normal volunteers. One normal volunteer's 2 mm biopsy was not obtained because he fainted after the 3 mm punch biopsy was taken.

**Table 1:** Initial Clinical Diagnosis, Results of Microbiology and Histopathology, Final Classification, and Procalcitonin Results.

ID	Clinical Diagnosis	Prior Antibiotics	Culture	PCR	Histopathology	Final Diagnosis	Procalcitonin (ng/mL)
010	Cellulitis	-	MRSA	+	3–4+ deep dermal neutrophilic infiltrate consistent with cellulitis; Gram positive intracellular cocci	Bacterial cellulitis	undetectable
013	Cellulitis	-	α-hemolytic strep, *Enterococcus* sp.	+	Stasis dermatitis with mixed acute and chronic inflammation; Gram stain negative.	Bacterial cellulitis	undetectable
015	Cellulitis	-	MSSA from pustule	-	Superficial and deep perivascular and interstitial (into subcutis) mixed infiltrate (neutrophils and lymphocytes) consistent with cellulitis; Gram stain equivocal/negative.	Bacterial cellulitis	undetectable
003	Cellulitis	-	-	-	Consistent with lymphocytic vasculitis. The biopsy shows a permeative cuff surrounding several deep dermal/subcutaneous vessels, as well as an overlying lymphocytic infiltrate surrounding the eccrine coil. Findings typical of bacterial cellulitis are lacking. Lymphocytic vasculitis may relate to a vasculopathic hypersensitivity reaction (e.g. due to a drug) or connective tissue disease. Clinical correlation required.	Inflamed control	0.13
002	Cellulitis	-	-	-	Superficial perivascular lymphocytic infiltrate with associated dermal edema and rare eosinophils: Gram stain negative (such findings, although non-specific, may be seen in association with a predominantly dermal delayed hypersensitivity reaction). Clinical correlation is required.	Inflamed control	undetectable
018	Cellulitis	2 days	-	-	Superficial dermal perivascular lymphocytic infiltrate with rare eosinophils and neutrophils consistent with mild hypersensitivity reaction (possibly mixed type IV and I); Gram stain negative.	Inflamed control	0.10
020	Dermatitis	-	-	-	Superficial and mid-dermal perivascular lymphocytic infiltrate with occasional eosinophils consistent with delayed hypersensitivity reaction; Gram stain negative.	Inflamed control	undetectable
021	Dermatitis	-	-	-	Dermal edema, superficial and deep, with sparse associated perivascular lymphoid component (non-specific). Gram stain negative.	Inflamed control	undetectable
026	Dermatitis	-	-	-	Fat only (no additional tissue in block). Gram stain negative.	Inflamed control	undetectable
031	Dermatitis	-	-	-	Superficial perivascular lymphocytic infiltrate with aggregated neutrophils in scale, most consistent with delayed hypersensitivity reaction, possibly impetiginized; Gram stain negative.	Inflamed control	undetectable
029	Dermatitis	-	coag-neg, non-hemolytic Staph	+	Superficial perivascular lymphocytic infiltrate most consistent with delayed hypersensitivity reaction; Gram stain negative.	Inflamed control	undetectable
007	Cellulitis	1 day	-	-	Superficial dermal edema and scattered superficial and deep dermal lymphocytes with rare admixed neutrophils. Gram stain negative (such findings are non-specific, although the possibility of a nearby or resolving area of more diagnostic cellulitis cannot be excluded).	Indeterminate	0.06
008	Cellulitis	3 days	-	-	Superficial and deep perivascular and interstitial mixed lymphocytic and neutrophilic infiltrate; Gram stain negative (the findings could indicate mild cellulitis; clinical correlation is required).	Indeterminate	0.06
009	Cellulitis	-	coag-neg, non-hemolytic Staph	-	Intralumenal neutrophils; consistent with urticarial immune response in appropriate clinical setting; Gram stain negative.	Indeterminate	undetectable
012	Cellulitis	<1 day	-	-	Minimal changes (no fat included); Gram stain negative.	Indeterminate	0.05
017	Cellulitis	-	-	-	Superficial dermal vascular prominence with basement membrane thickening (? stasis, age-related, diabetes, hypertension); Gram stain negative.	Indeterminate	undetectable
022	Cellulitis	-	-	-	Superficial and deep perivascular mixed inflammatory infiltrate, neutrophil predominant, consistent with cellulitis in appropriate clinical setting. Gram stain: no definitive bacteria seen.	Indeterminate	0.37
023	Cellulitis	-	-	-	Superficial and deep sparse perivascular and interstitial mixed infiltrate approx. 50:50 neutrophils to lymphocytes);? Cellulitis; Gram stain- rare Gram positive (extracellular) of uncertain significance at tissue edge.	Indeterminate	undetectable
027	Cellulitis	-	-	-	Superficial and deep (incl. subcutis) mixed inflammatory infiltrate containing neutrophils, consistent with cellulitis; Gram stain negative.	Indeterminate	undetectable
028	Cellulitis	2 weeks (for another indication)	coag-neg, non-hemolytic Staph	-	Superficial and deep sparse perivascular lymphocytic infiltrate (non-specific); Gram stain negative. *Note*: *this patient was diagnosed as having a cutaneous allergy to an antibiotic given for another indication*.	Indeterminate	undetectable
019	Dermatitis	-	α-hemolytic strep, corynebacteria	-	Evolving lichen simplex chronicus with surface impetiginization	Indeterminate	undetectable
001	Normal volunteer	-	-		Sparse superficial perivascular lymphocytic infiltrate; Gram stain negative (potentially within normal limits)	Normal	undetectable
005	Normal volunteer	-	-	-	Within normal limits; Gram stain negative.	Normal	undetectable
006	Normal volunteer	-	-	-	Within normal limits; Gram stain negative.	Normal	undetectable
016	Normal volunteer	-	-	-	Within normal limits; Gram stain negative.	Normal	undetectable
030	Normal volunteer	-	Not collected	Within normal limits; Gram stain negative.	Normal	undetectable
032	Normal volunteer	-	-	-	Within normal limits; Gram stain negative.	Normal	undetectable
033	Normal volunteer	-	-	-	Within normal limits; Gram stain negative.	Normal	undetectable

S1 Table, available online, presents additional provides additional information on the participants, namely age, sex, reason for ED visit, eGFR (estimated glomerular filtration rate), diabetes, and other comorbidities.

### Microbiology Results

Of 27 biopsies of normal skin, five had positive cultures. Four were skin commensals (corynebacteria and non-hemolytic coagulase-negative staphylococci), and one was a non-pathogenic *Bacillus* species, all presumed to be contaminants. PCR was positive in only one of the 28, which was also culture-positive for a commensal.

**Fig 5:**
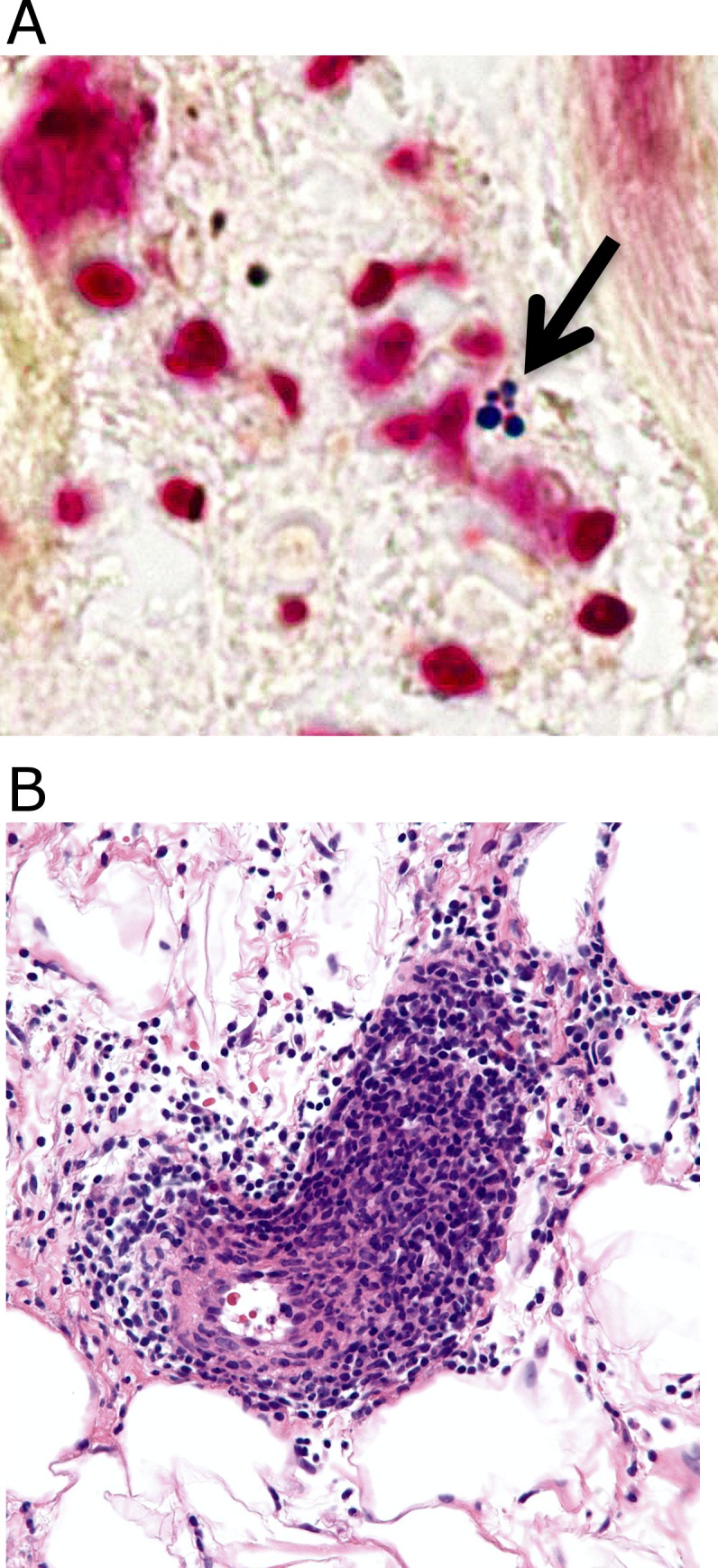
Photomicrographs from patients diagnosed clinically as having bacterial cellulitis. Fig 5A: Culture-proven MRSA cellulitis with positive Gram stain. Note intracellular Gram-positive cocci in clusters. Fig 5B: Lymphocytic vasculitis misdiagnosed as cellulitis. Note abundant lymphocytes invading vessel wall.

Of the 15 patients diagnosed clinically as having cellulitis, three (20%) had the diagnosis of bacterial cellulitis confirmed microbiologically, and were designated confirmed bacterial cases. One grew MRSA and was PCR-positive, and had a positive Gram stain (Fig 5A). The second grew α-hemolytic streptococci and an *Enterococcus* species, and was PCR-positive; histopathology was unremarkable (Table 1). The third grew MSSA from a pustule; histopathology was unremarkable.

Of the 6 patients with clinically-diagnosed non-infectious dermatitis, one was classified as indeterminate due to culture-positivity for α-hemolytic streptococci (Fig 1). The remainder had negative cultures and PCR, except one that grew a commensal (non-hemolytic coagulase-negative staphylococci, case 29). Thus, these patients contributed five inflamed controls and one indeterminate.

No ß-hemolytic streptococci were isolated in this study.

### Histopathology Results

Histopathology results are presented in Table 1. Most of the results were non-diagnostic, with nonspecific lymphocytic infiltrates or normal findings. However, of the 15 patients diagnosed clinically as having bacterial cellulitis, three (20%) had affirmative alternative histopathological diagnoses. One was a case of lymphocytic vasculitis (Fig 5B), and the other two were hypersensitivity reactions with eosinophilic infiltrates. Of these, all had negative cultures and PCR and were, therefore, classified as inflamed controls, in accordance with the algorithm shown in Fig 1. Adding these to the five inflamed controls derived from patients with clinical diagnoses of non-infectious skin inflammation (see above) brought the total to eight inflamed controls. In summary, our algorithm classified the 28 participants as follows: three verified bacterial cellulitis cases, eight verified sterile inflamed controls, ten indeterminate participants, and seven normal volunteers.

### Procalcitonin Results

All three of our confirmed cellulitis cases had undetectable serum procalcitonin levels. This suggests a sensitivity of 0% for procalcitonin as a diagnostic test for bacterial cellulitis, although we refrain from calculating formal test characteristics (e.g., c-statistic) due to the small sample size of this pilot study. In tissue and blood, expression of the CALCA gene did not differ in cases vs. inflamed controls (data not shown). Blood levels <0.5 ng/mL are considered negative, even if detectable, and all of the remaining samples were negative as well, although some had results above the threshold of detection (Table 1).

### Immunomodulator Gene Expression Results

Of 595 human mRNA molecules,[19] the one with the highest differential expression in the blood of cases vs. inflamed controls was HLA-DQA1, whose expression was 34 times higher in cases than in inflamed controls (geometric mean of 2427 vs. 71 counts per 100 ng RNA, p = 0.009). HLA-DQB1, the other component of the heterodimer encoded by HLA-DQA1, was expressed 17-fold more in cases (p = 0.007). In lesional tissue, as in blood, HLA-DQA1 was more highly expressed in cases than in inflamed controls, with a geometric mean fold-change of 18.5 (p = 0.04).

We then selected all mRNA molecules whose differential expression had a p-value ≤0.01 and used them to generate the heat map shown in Fig 3. This reveals a distinctive profile of gene expression for the three bacterial cases (leftmost columns). Two of the inflamed controls (far right) have a somewhat similar expression profile to that of the cases, but visual inspection suggests that their gene expression patterns were different enough that a quantitative model would be able to distinguish them from the cases. In the worst case scenario, these would not be distinguishable; that would imply that this immunologic signature would be *sensitive* for detection of cellulitis but not *specific*. The practical application of an assay that might result from this is that the immunological signature might be used to *rule out* bacterial infection in a patient suspected of having cellulitis. It is interesting to note that the gene expression profile of the three participants who had a clinical diagnosis of cellulitis but a gold-standard diagnosis of non-infectious dermatitis are the first three inflamed controls (leftmost “control” columns): their gene expression profiles are more similar to the profiles of the inflamed controls than those of the cases, suggesting that the algorithm categorized the participants correctly.

Fig 4 shows a heat map that compares log2-transformed gene expression in each of the three comparison groups to that in verified bacterial cases. It suggests that gene expression in the verified cases was different from expression in the three comparison groups, and that the contrast was similar across the three comparisons. This suggests, in turn, that expression of these genes in normal volunteers and patients with sterile lesions is similar, and systematically different from the gene expression seen in the verified bacterial cases. This would seem to validate our algorithm, while providing evidence that the “cellulitis” patients with sterile lesions were more similar to “dermatitis” patients and normal volunteers than to the confirmed cases of bacterial cellulitis.

## Discussion

In this pilot study of 28 participants, we implemented a novel classification algorithm, which allows an investigator to identify verified bacterial cellulitis cases and inflamed controls using affirmative microbiological and histopathological results (Fig 1). Of 15 patients diagnosed clinically as having bacterial cellulitis, only three (20%) had pathogens identified in biopsy specimens. This is not surprising, because many prior studies have sought to identify bacterial pathogens in cellulitis specimens and no study has succeeded in identifying pathogens in even 30%.[11–13] (The studies by Bernard et al. in the 1980s should not be considered here, because they were studies of morphologically-typical erysipelas and necrotizing fasciitis, rather than garden-variety cellulitis.[22,23])

We isolated no ß-hemolytic streptococci. This is harmonious with another recent study, that also found no ß-hemolytic streptococci in a sample of 50 subjects diagnosed as having cellulitis.[24] Other evidence casts doubt on the traditional notion that cellulitis is a streptococcal disease: a review of 16 studies from 1966–2007 using needle aspiration or biopsy to identify pathogens in cellulitis found that only 16% of cellulitis subjects had positive cultures, and of these, a minority were streptococcal while a majority were staphylococcal.[13] Serological evidence for streptococcal infection with negative culture or PCR is conflicting.[25,26]

Although statistical power was obviously limited, the pilot study yielded disappointing results regarding procalcitonin as a diagnostic test: this marker was undetectable in the confirmed bacterial cases. There is broad recognition that procalcitonin is a useful diagnostic tool to rule in or out bacterial infection in a variety of conditions, but our findings are consistent with the manufacturer’s label, warning that serum procalcitonin may not be elevated in by localized infection.[14,27–29]

We used the new classification algorithm to search for biomarkers capable of differentiating cases from inflamed controls, via transcriptomics. HLA-DQA1 was expressed 34-fold more in cases than in inflamed controls. Our investigation is limited by the small sample size and we have refrained from calculating test characteristics for this or other markers. We view our pilot findings as hypothesis generating, rather than hypothesis testing. Nevertheless, there is biological plausibility to the concept of HLA-DQA1 as a marker of bacterial infection. This moiety is part of the heterodimer used by antigen presenting cells to display foreign peptides to T cells. It is plausible that its expression would be activated by bacterial infection, and thus it may be a potential biomarker, though the methodology to develop clinically-actionable information remains to be defined.

Finally, we demonstrated methods for development of a multivariable immunological signature of bacterial skin infection. Due to the small sample size of this pilot study, we limited ourselves to a qualitative analysis, via heat maps indicating patterns of gene expression. The analysis suggests that it may be possible to identify a panel of biomarkers that can serve as a sensitive signal of bacterial infection, as our confirmed bacterial cases had a pattern of gene expression that differed consistently from the expression patterns in the three comparison groups (Fig 4).

A practical lesson from this study is that cultures of normal skin biopsies rarely harbor pathogens after preparation with three alcohol swabs, without betadine or chlorhexidine. As we plan our next studies, this finding suggests that normal volunteers may not be necessary as controls.

## Limitations

This was a pilot study, designed to test the new algorithm (Fig 1) and the multidisciplinary methods used. The small sample precluded quantitative statistical analysis, and we emphasize that the findings are hypothesis generating. Another limitation is that, due to resource constraints, we did not measure acute and convalescent streptococcal serologies. As noted above, serological evidence for a streptococcal etiology of cellulitis is conflicting.[25,26] In a planned larger study, we will add serological classification to the algorithm shown in Fig 1, and this may result in some of the “indeterminate” lesions being classified as confirmed infected cases. For example, perhaps the rightmost controls in Fig 3 might have positive serological evidence of a streptococcal infection.

## Conclusion

In this pilot study, we assembled a multidisciplinary team to accomplish the first study of cellulitis to include diagnostic histopathology and to compare confirmed inflamed cases to confirmed non-infected inflamed controls. We developed an algorithm that can serve as a gold standard for classification of skin inflammation lesions in research. We classified patients and assessed the sensitivity of procalcitonin. The procalcitonin results were disappointing, with undetectable blood levels in microbiologically-proven bacterial cellulitis cases. We also piloted the use of transcriptomic analysis of tissue and blood, finding a distinct immunological signature of confirmed bacterial cellulitis relative to confirmed non-infected controls, relative to indeterminate inflamed lesions, and relative to normal volunteers. With a larger sample, we plan to use data reduction methods to eliminate co-linear predictors, and identify a set of markers that can be used to generate a score–a quantitative test for bacterial infection. The implications of this pilot study for future research are that the algorithm we have developed may be used to develop experiments that compare confirmed infected cases to confirmed sterile inflamed controls, instead of the uncontrolled case series that have been published heretofore. The ultimate clinical import of this line of research is that it may be possible to develop a multi-marker panel that can reliably rule out bacterial infection, which would allow clinicians to refrain from prescribing antibiotics and encourage them to search for etiologies other than bacterial infection, such as venous stasis dermatitis, contact dermatitis, or vasculitis.

## Supporting Information

S1 Table Demographic and Clinical Data. (DOCX)
