# Supplementary material for: Toward an Objective Diagnostic Test for Bacterial Cellulitis
Source: PLoS One. 2016 Sep 22;11(9):e0162947. doi: 10.1371/journal.pone.0162947 (PMC5033594; doi:10.1371/journal.pone.0162947)
Supplement: S1 Table — (DOCX) [file pone.0162947.s001.docx]

**Supplementary Table: Demographic and Clinical Data**

| *ID* | *Clinical Diagnosis* | *Final  Diagnosis* | *Age (years)* | *Sex* | *Reason for ED Visit* | *Last eGFR* | *Diabetes* | *Other Comorbidities* |
| --- | --- | --- | --- | --- | --- | --- | --- | --- |
| 010 | Cellulitis | Bacterial cellulitis | 59 | F | Leg pain | >59 | No | Uterine cancer, chemotherapy 5 weeks ago, chronic lower extremity edema, drug abuse |
| 013 | Cellulitis | Bacterial cellulitis | 74 | M | Leg infection | >59 | Yes | Chronic lymphedema in affected leg, atrial fibrillation on Coumadin |
| 015 | Cellulitis | Bacterial cellulitis | 21 | F | Infected wound | >59 | No | Eczema, recently biopsied, biopsy site became infected. |
| 003 | Cellulitis | Inflamed control | 50 | M | Fever, leg redness | 33 | Yes | Obesity, peripheral neuropathy, substance abuse, depression, sleep apnea, asthma, CHF, gout |
| 002 | Cellulitis | Inflamed control | 71 | F | Leg swelling | >59 | Yes | Hypertension, asthma, osteoporosis, migraines |
| 018 | Cellulitis | Inflamed control | 77 | F | Weakness | 56 | No | Hypertension, depression, meningioma, zoster, hyperthyroidism, hypercholesterolemia, anxiety, osteopenia |
| 020 | Dermatitis | Inflamed control | 24 | F | Skin bumps | >59 | No | None |
| 021 | Dermatitis | Inflamed control | 70 | M | Shortness of breath, leg swelling | >59 | Yes | CHF, aortic insufficiency, atrial fibrillation not on Coumadin, strokes, DVT, aortic aneurysm, hypertension, dementia |
| 026 | Dermatitis | Inflamed control | 25 | F | Eczema flare | – | No | Eczema, otherwise healthy |
| 031 | Dermatitis | Inflamed control | 70 | M | Syncope | 55 | No | Hypertension, gout |
| 029 | Dermatitis | Inflamed control | 31 | F | Rash | – | – | – |
| 007 | Cellulitis | Indeterminate | 45 | M | “Cellulitis” | >59 | Yes | Already on treatment for leg cellulitis, now worsening. Obese, history of necrotizing fasciitis, hypertension, hyperlipidemia. |
| 008 | Cellulitis | Indeterminate | 51 | F | Right-sided abdominal pain | >59 | No | Cardiomyopathy, valvuloplasty, stroke, myocardial infarction, hernia repair, presenting with vomiting associated with an antibiotic given for a questionable cellulitis lesion on the abdomen |
| 009 | Cellulitis | Indeterminate | 23 | F | Insect bite | – | No | None |
| 012 | Cellulitis | Indeterminate | 70 | M | Leg redness | >59 | No | Gastric cancer not currently under treatment, hypertension, hyperlipidemia, gout, coronary artery disease, atrial fibrillation |
| 017 | Cellulitis | Indeterminate | 63 | F | Leg swelling | >59 | Yes | Hypertension, hypercholesterolemia |
| 022 | Cellulitis | Indeterminate | 19 | M | Leg pain | >59 | No | None |
| 023 | Cellulitis | Indeterminate | 28 | F | Thigh swelling | >59 | No | Bacterial vaginosis |
| 027 | Cellulitis | Indeterminate | 26 | F | Arm redness | >59 | No | Grave’s disease |
| 028 | Cellulitis | Indeterminate | 29 | M | Insect bite | >59 | No | None |
| 019 | Dermatitis | Indeterminate | 71 | F | Rash | >59 | No | Ulcerative colitis, gastroesophageal reflux |
| 001 | Normal  volunteer | Normal | 27 | M | – | >59 | No | None |
| 005 | Normal  volunteer | Normal | 36 | M | – | – | No | None |
| 006 | Normal  volunteer | Normal | 43 | M | – | – | No | None |
| 016 | Normal  volunteer | Normal | 24 | F | – | – | No | None |
| 030 | Normal  volunteer | Normal | 26 | M | – | – | No | None |
| 032 | Normal  volunteer | Normal | 24 | F | – | – | No | None |
| 033 | Normal  volunteer | Normal | 25 | M | – | – | No | None |
